# Globalizing queer? AIDS, homophobia and the politics of sexual identity in India

**DOI:** 10.1186/1744-8603-3-8

**Published:** 2007-07-11

**Authors:** Subir K Kole

**Affiliations:** 1 Degree Fellow, East-West Center, Lecturer, Department of Political Science, University of Hawaii at Manoa, 1711 East-West Road, MSC 836, Honolulu, HI 96848, USA

## Abstract

Queerness is now global. Many emerging economies of the global South are experiencing queer mobilization and sexual identity politics raising fundamental questions of citizenship and human rights on the one hand; and discourses of nationalism, cultural identity, imperialism, tradition and family-values on the other. While some researchers argue that with economic globalization in the developing world, a Western, hegemonic notion of lesbian, gay, bisexual and transgender (LGBT) identity has been exported to traditional societies thereby destroying indigenous sexual cultures and diversities, other scholars do not consider globalization as a significant factor in global queer mobilization and sexual identity politics. This paper aims at exploring the debate around globalization and contemporary queer politics in developing world with special reference to India. After briefly tracing the history of sexual identity politics, this paper examines the process of queer mobilization in relation to emergence of HIV/AIDS epidemic and forces of neoliberal globalization. I argue that the twin-process of globalization and AIDS epidemic has significantly influenced the mobilization of queer communities, while simultaneously strengthening right wing "homophobic" discourses of heterosexist nationalism in India.

## Background

"Queerness is *now *global. Whether in advertising, film, performing arts, the internet or the political discourses of human rights in emerging democracies, images of queer sexualities and cultures *now *circulate around the globe" [1]. While there is no reason to deny that queerness *is *indeed global, the phrase "now" in the above sentence indicates that it was not global earlier. Understood this way, one can logically ask, if queerness has *now *gone global, which brand of it has been globalized?

In recent years, India has witnessed a growing activism of various NGOs and civil society institutions toward mainstreaming sexually minority groups. Such efforts toward mainstreaming consist of advocating the rights of lesbian, gay, bisexual and transgender groups (henceforth LGBTs), campaigning against laws that discriminate their rights, seeking public petition for withdrawal of such laws, and efforts to normalize the recognition and acceptance of LGBT identity categories in India. Contrary to this activism, a large section of Indian society believes that such efforts of mainstreaming pose a threat to the social and cultural integrity as well as moral fabric of Indian nation. Believers of this ideology include both left and right, Marxist thinkers as well as right wing radical Hindu nationalist groups, and a major part of functioning Indian bureaucracy, including a huge segment of its 700 million rural population. This paper aims to capture the debate around mobilization of queer communities for their civil and political rights and analyze the emerging politics of sexual identity in relation to globalization and HIV/AIDS epidemic in the Indian subcontinent.

In every culture and society, throughout history, people have attempted or practiced every anatomically possible form of sexual stimulation and gratification. Hardly any of these practices have ever become the question of sexual identity politics. The differences in patterns of sexual expression among societies derive from their history, culture, present circumstances and power relations that determine whether their actual patterns of sexual behavior remain open or hidden. The best person to theorize this is Michael Foucault who noted that "the homosexual" became a "species" circa 1870 in an epoch of Western society that relied upon an urge to confess sexual practice as a means to uncover a "truth" in "human nature" [2]. Thus not confessing one's sexual practice and the discursive rubric of taboo and repression prevented access to personal "truth." Though homosexuality as a practice has been in existence in traditional societies since time immemorial, sexual identity has never become an agenda of political struggle in any of these societies until recently. For example, many individuals in India or other traditional societies may practice same-sex sexual relations but do not identify themselves as "gay" or "lesbian." For many men in India, having same-sex sexual relations is equal to *masti *or having fun, and they refuse to be identified as "gay" [3-5].

Thus, though homosexual behavior (the act of sodomy), not identities (such as gay or lesbian), remains a "criminal offence" under Section 377 of the Indian Penal Code (IPC), historically, Indian society acknowledges and tolerates certain degree of homosexual behavior between consenting adults in private. Even the Government of India acknowledges through an affidavit submitted to Delhi High Court in response to a public petition challenging the constitutional validity of IPC 377 that, "the state will turn a blind eye if homosexuality is practiced between two consenting adults in private" [6]. The issue has become sensitive in a sexually conservative society like India with sexually minority groups challenging the public/private boundary and the authority of the State to make laws that discriminate their rights. While some researchers [4,7-10] contend that with economic globalization in the developing world, a Western, hegemonic notion of LGBT identity has been exported to traditional societies thereby destroying indigenous sexual cultures and diversities, other scholars [11] do not consider globalization as a significant factor for global queer mobilization and sexual identity politics. Yet, a cursory look at the present cartography of the globe reveals that countries where LGBT identities are now emerging broadly correspond with global-South that have recently opened up their economies to neoliberal capital by adopting IMF-sponsored structural adjustment program wherein homosexuality still remains "illegal" [12]. How does then one conceptualize the North-South/East-West divide and explain emerging politics of sexual identity in newly globalizing economies?

Central to the above question is the notion of a "discourse" around human sexuality and the "truth" and "power" that were produced through such discourses in postmodern, postindustrial, capitalistic societies of the West. Sexual and gender plurality, sexual preference, sexual identity and "coming out" thus became an important indicator of a so called "developed" society. Traditional societies that could not capture these modern notions of sexual identity categories were considered "inferior," "sexually repressed" and hence need to be "developed" and "freed" thereby necessitating an intervention from outside. Any resistance to these efforts of liberation was considered as "homophobia" and all traditional, non-modern societies thus came to be known as "homophobic" societies in which sexual minorities require liberation. Under the present world economic and social order, such intervention of liberating sexually repressed communities in traditional homophobic societies takes place through Western institutions of international development, aid agencies, donor organizations and international NGOs. With reference to international development, Escobar (1984, 1995) noted that the "Third World" was actually invented by the West through discourses of (under)development, and this discovery created a field of intervention through which developed countries and their associated institutions exercised tremendous "power" over the Third World [13,14]. This paper examines what happens when Western donor discourses help the East uncover their "repressed" sexualities primarily through local subjects and NGOs working on sexuality and HIV/AIDS prevention. Following Shannon Woodcock (2004), I contend that India has a diverse, complex and elaborate spectrum of same-sex sexual cultures in which sexual minorities have always performed their identities in a variety of ways, in a variety of social spaces and *without *the political rhetoric of the West. The Western project of liberating the "sexually repressed" communities of the East attempts to contain this dynamic and diverse sexual culture by baptizing traditional sexual minorities to evolve into a globalized, universal, and totalizing LGBT identity category.

In this paper, I use the term LGBTs only to refer to the modern/postmodern context of emerging sexual identity categories, and not to denote any traditional sexually minority groups/identities that predated its existence. By this conceptualization, *hijras, kothis, kinnars, panthis, jogtas, dangas, alis, double-deckers, chhakkas, dhuranis *and any other indigenous communities who identify and relate themselves by sexual practices would not be considered as LGBTs, though, they are commonly referred to as such in most HIV/AIDS and sexuality discourses in India. To avoid this complexity, "queer" is preferred over other terms (though not commonly used in India) by many activists and individuals since it does not confine sexual identities in fixed LGBT categories and allow for much space and ambiguities for diverse sexualities to be included. Queer encompasses a multiplicity of desires and diverse sexualities outside the homo/heterosexual matrix in which *identity *is seen as performative, something that we do and act out rather than possessing it, and something that we assemble from existing discursive practices [15,16]. Historically used as a derogatory term to refer to homosexual people in the West, queer was later reclaimed by theorists and activists to refer to multitude of subject positions that question the naturalness, rightness and inevitability of heterosexuality. "Queer/ness," thus, by its very nature of inclusiveness, can be viewed as another concept that by way of encompassing every possible sexual diversities in one single fold, attempts to obscure spatial and temporal differences in multiple sexual subject positions.

This paper is organized into following three sections: in the first section, I briefly review the history of emerging sexual identity politics in India and some of the recent movements and grassroots activism of various NGOs and civil society institutions toward mainstreaming sexual minority groups. In second part, I trace the origin of such activism in relation to globalization and emerging AIDS pandemic in Indian subcontinent. Section three of this paper examines the implications of a donor-driven LGBT politics in Indian social and cultural context. Based on general arguments, section four draws basic conclusions.

At this point, it is important to make clear what is not up for discussion here. This paper is not an addition to the existing literature about how sexual minorities are generally oppressed in the society due to prejudice and stigma and how the legal provision discriminates their basic human rights as citizens of India [17-21]. This paper is also not about explicating complex theoretical strands and feminist critique of "gender," "power" and "performance" advanced by some of the important queer theorists as Judith Butler and Eve Kosofsky Sedgwick; or examining theoretical works on queer diasporas, postcolonial queer subjectivities and queer representation in the media [22-32]. I also do not intend to deal with everyday implications of Section 377 of IPC on queer communities, or the genuine need that this colonial, draconian law indeed deserves to be repealed in its own merit [33-36]. And finally, this paper is also not about any policy recommendation or future course of action for LGBT rights [18,19,36,37]. Considering all these, one may question that the present exercise is narrowly focused, which is intentional and which I think, is essential for maintaining clarity. Among various other factors contributing to queer mobilization in India, such as capitalist modernization, discourses of universal human rights, new social movements, resistance to dominant power structures, and evolving democracy and minority rights [33] (p. 66–68), I only examine two important factors of Western donor and local NGO discourses on sexual rights and looming HIV/AIDS epidemic in India; and the ways in which these two processes have been mediated through globalization to influence the LBGT/queer identity politics in India. While doing so, I duly acknowledge that there are several individual efforts, informal support groups, collectives and "agency" of indigenous queer communities that operate outside HIV/AIDS/sexuality funding. However, these efforts, though commendable, are not part of my discussion.

### 1. Tracing the history of LGBT identity politics

The phenomena of confessing one's sexual identity as a means to uncover personal "truth" is relatively recent in India and the "out" LGBTs were not visible in the country until 1990s. Though writings of romantic same-sex love stories, Urdu poetries and *ghazals *could be traced back in pre-independent India, writers of such novels or stories hardly ever confessed their sexual identity publicly. For example, India's celebrated poet Firaq Gorakhpuri (1896–1982) or a Bengali literary giant Michael Madhusudhan Dutt (1824–1873) who were known to be homosexual through their writings, never identified themselves as such. Pandey Bechan Sharma's *Chocolate *(1927), and Ismat Chugtai's *Lihaaf *(The Quilt 1942), though based on homoerotic love stories and both these novels drew widespread public attention and protest including lawsuit, the authors never claimed homosexuality as their identity [32].

In later years, such as in Rajkamal Chaudhury's Hindi novel *Machhli Mari Hui *(Dead Fish 1965), same sex relationship between men and women has been represented as something imported from the West (US) and a symptom of capitalism and neo-colonialism [38]. Kamala Das who wrote an autobiographical account *My Story *(1976), depicting her extramarital affair, her adolescent crush on a female teacher, and a brief lesbian encounter with an elder student, is still not considered as a lesbian writer. More recently Shobha De's *Strange Obsession *(1993), considered as a soft-porn in the literary circle deals with a lesbian affair where the heroin is rescued by marriage. Shobha De, the mother of six children and married to a very wealthy Mumbai businessman is not considered a lesbian writer. The first academic book on Indian homosexuals appeared in 1977 (*The World of Homosexuals*) written by Shakuntala Devi, the mathematics wizkid who was internationally known as the *human computer*. This book treated homosexuality in a positive light and reviewed socio-cultural and legal situation of homosexuality in India and contrasted that with the then gay liberation movement in USA [39].

Quite a contrary trend is observed in late 1980s-India or more specifically in late 1990s, when authors dealing with the subject of homosexuality "came out" with their sexual identity through their writing. A large part of this "confession" took place in the preface, introduction or acknowledgement section of their books. This revolution started with authors and film makers of Indian origin who were born and brought up in the West and had successfully established themselves in western academic and professional world. Most important among them were the works of Suniti Namjoshi (*The Conversations of Cow 1985; Because of India 1989*); Pratibha Parmar (*Khush 1991; Queer Looks 1993); *Rakesh Ratti (*A Lotus of Another Color 1993*) from India and Shyam Selvadurai (*Funny Boy 1994; Cinnamon Gardens 1999*) from Sri Lanka. Summers (1995) point out that the relative openness of this small group of writers was perhaps largely due to their diasporic locations. They live in either the United States or Britain, countries that have well-established gay and lesbian communities with a tradition of organized resistance and therefore have greater sexual and artistic freedom and wider publishing opportunities [40]. Further, their physical separation from family and community probably gives them relative privacy and greater freedom from culturally imposed constraints.

Since mid-1980s, hundreds of young gay and lesbian South Asians living in metropolitan centers of Europe and North America have begun to assert their presence by forming support groups, begun partly in response to the racism they encounter in predominantly white queer communities of the West (Summers 1995). Many of the groups regularly publish newsletters, such as *Shakti Khabar *(London), *Trikone *(San Jose), *Shamakami *(San Francisco), and *Khush Khayal *(Toronto), which have subscribers in many countries of South Asia. These publications seek to link South Asian gay and lesbian individuals as well as communities scattered around the world and to help forge a global South Asian queer identity.

The "confessional" tradition set by South Asian queer diasporic communities influenced writers from India. Some of the important recent authors include Giti Thadani (*Sakhiyani: Lesbian Desire in Ancient and Modern India, 1996); *Ashwini Sukthanker (*Facing the Mirror 1999); *Hoshang Merchant (*Yaraana: Gay writing from India 1999); *and later Salim Kidwai and Ruth Vanita (*Same Sex Love 2000*, and *Love's Rite 2005*). After globalization, trade liberalization, and opening of Indian economy to foreign direct investment in 1991, the process of "confession" has become more overt from writing to political action and assertion of one's own identity and demand for a queer-space. The pace at which such a development took place, can indeed be called a revolution.

#### 1.1. Mainstreaming sexual minorities: Initial years

Some unorganized initial efforts to bring forth the issues of sexual minorities in India could be traced back in 1990. In 1990, India's first exclusive gay magazine, *Bombay Dost *(Bombay Friends) was published by an "out" gay journalist Ashok Row Kavi, who later in 1994 established his own NGO, Humsafar Trust to work with LGBT groups in Mumbai. *Bombay Dost *was a small newsletter of gay men initially published intermittently in Hindi until 1994 through which they tried to establish local networks of gay groups and provide information to men who have sex with men (henceforth MSMs). Since late 1994, *Bombay Dost *has become an exclusively English language magazine serving upper class, educated elites within urban India. It seems that probably enough number of Hindi readers were not available. The class-bias is also reflected from pricing structure of the magazine. A single copy in 1994 used to cost Rs. 40, which was equivalent to the total earning of a daily wage laborer. It may also be due to low economy of scale that the price of an individual copy went up. In either case, *Bombay Dost *did not serve the marginalized, lower class sexual minorities in India. Moreover, a review of the magazine over the last decade reveals that much attention was paid on featuring international gay news and issues that would possibly have little relevance to Indian gays.

In 1991, a human rights activist group, *AIDS Bhedbhav Virodhi Andolan *(Anti-AIDS Discrimination Movement) known as ABVA published its first report *Less than Gay: A Citizens' Report on the Status of Homosexuality in India*. Through this report, the ABVA advocated for civil rights of LGBTs to include same sex marriage, parenting, decriminalization of homosexuality and repeal of IPC 377, amendments in Special Marriage Act and AIDS Prevention Bill of 1989, and providing a positive homosexuality education in school [36] (p. 92–93). In 1994, ABVA reported that there is incidence of rampant homosexuality in Tihar jail of New Delhi and recommended the jail authorities that condoms to be made available to prison inmates for preventing HIV transmission. The Inspector General of Prisons (the then Magsaysay Award winner, Kiran Bedi) refused to agree with the plea on the ground that distributing condoms would mean that government is promoting homosexuality in prison by violating law of the land, Section 377 IPC (Unnatural Offences). The law reads: *"Whoever voluntarily has carnal intercourse against the order of nature with any man, woman, or animal, shall be punished with imprisonment for life, or with imprisonment of either description for a term which may extend to ten years, and shall also be liable to fine" *[35] (p. 5). Though this law enacted by the British in 1861, does not differentiate between homosexual act and identity, a person can not be sentenced under this law who claims his sexual identity as gay, but act/behavior is not proved. In 1994, ABVA first challenged the constitutional validity of Section 377 IPC in Delhi High Court. Through its petition, ABVA argued for supplying of condoms to jail inmates and instructing the authorities to refrain from segregating prisoners with homosexual orientation or those suffering from HIV/AIDS. The petition argued that Section 377 should be repealed because it violates the right to privacy and discriminates against people with a particular sexual orientation. Even after 13 odd years, the case is still pending with Delhi High Court, despite the fact that the Law Commission of India in its 172^nd ^Report (2000) has already recommended repealing Section 377 of IPC [41].

The emergence of looming AIDS epidemic in Indian subcontinent and economic globalization of early 1990s influenced queer mobilization and queer movement in some fundamental ways. From earlier sporadic and individual efforts of early 1990s, the struggle against law and the process of queer mobilization shifted toward a more donor driven and AIDS-induced agenda (though simultaneously, individual and collective level efforts have multiplied during the same period). A large part of queer mobilization took place in response to HIV epidemic and due to vulnerability of some queer people resulting from their behavioral aspect. NGOs working with sexually minority groups have largely mobilized a diverse spectrum of indigenous queer sexualities under a fixed banner of "LGBT identities," though the queers continue to identify themselves as *hijras *or *kothis*. The following section examines this process in a historical context of globalization and AIDS epidemic in India.

### 2. Globalization and a decade of LGBT activism

Is it just a mere coincidence that the emergence of LGBT activism broadly corresponds with two important landmarks of economic and social history of India? I refer to these two landmarks as: first, opening up of Indian economy in 1991 and adopting IMF-sponsored structural adjustment program of promoting free trade and free market regime; and second, the looming presence of HIV/AIDS epidemic in Indian subcontinent and thereby accepting World Bank loan for prevention and control of AIDS in India.

#### 2.1. Structural adjustment program (SAP)

While the root cause of structural adjustment and economic reform process could be traced back to Third World Debt Crisis of 1980s, the economic liberalization in question dates from July 1991. During 1990–91, India experienced one of the worst years in its economic, social and political history. It was marked by political instability with frequent changes in government, increasing budget deficit, falling foreign exchange reserve and shooting inflation of up to 17 percent. Between December 2, 1989 to June 21, 1991, India experienced change of four governments at the Centre led by Rajiv Gandhi, V.P. Singh, Chandra Sekhar, and P.V. Narsimha Rao [42]. Toward the end of June 1991, India's foreign exchange reserve drastically fell to almost $1 billion, less than sustaining 15 days of import bill. In the international market, credit rating for India plummeted. Access to international credit from private and commercial sources was closed. In July 1991, 47 tonnes of gold from the reserve assets of the Reserve Bank of India was shipped to the vaults of the Bank of England in a dramatic bid to raise $405 million from Bank of England and Bank of Japan [43], still in vein to tackle the economic crisis. The IMF agreed to give loan and rescue the government out of crisis provided India accepts its SAP. Ravaged by severe economic depression, the Parliamentary Standing Committee recommended the government to open up the economy and adopt a structural adjustment program.

**Figure 1:**
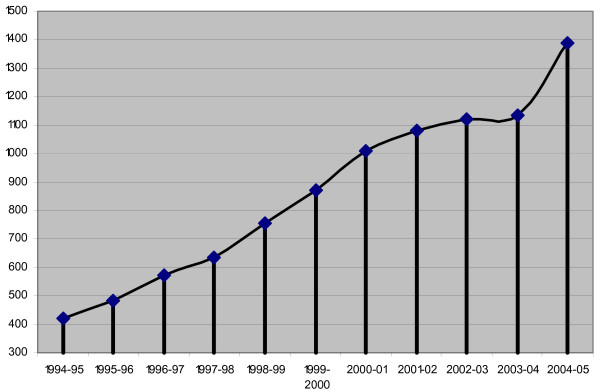
India: Receipt of Foreign Contribution by NGOs 1994-2005 (Million US Dollar), Source: FCRA Annual Report, MHA, Govt. of India, 2004, 2005.

By adopting such a historic measure, India tried to take advantage of economic globalization by promoting free trade and free market regime. On the contrary, giant players in the global economy tried to take advantage of vast unexplored Indian market. Thus with multinational and transnational corporations (such as LG, Samsung, Pepsi, and McDonalds), came the multinational and transnational NGOs (such as MSF, FXB, Pathfinder International, Engender Health, McArthur Foundation, ICRW, HIVOS, and in recent years Bill Gates, International HIV/AIDS alliance, Packard Foundation and about a hundred others). During last decade (1994–2005), the largest number of multinational NGOs entered in India. In the same logic of globalization as capital moves from capital-rich to capital-scarce areas in search of higher marginal return, the NGOs moved from the West to the East to work with newer communities. The exact number of how many such NGOs entered India after 1991, is difficult to estimate. However, in real terms, foreign contribution received by registered NGOs under Foreign Contribution Regulation Act (FCRA) increased from 420.5 million US dollars in 1994 to 1390.4 million US dollars in 2005 (Fig. 1), an increase of 230 percent over the last decade or almost 23 percent increase per annum [44]. The list of donor countries is headed by the US followed by Germany and UK whereas list of donors are headed by Ford Foundation, World Vision International and Christian Children's Fund (*ibid*.).

#### 2.2. Indigenous discourse on sexuality and AIDS

In 1991, with the initiative of an Indian HIV/AIDS activist in London, Shivananda Khan, the Naz Project came up to address the sexual health needs of queer South Asian communities in London. Though Khan was initially involved in organizing lesbian and gay support groups for people of South Asian origin between 1988–91, his primary work has remained HIV/AIDS and sexual health firstly in the UK and then in South Asian countries of Bangladesh, India, Nepal and Pakistan. In 1994, the NAZ Project (in association with its local organizer, the Humsafar Trust, Mumbai) sponsored the first national conference for gay-identified men and MSMs in South Asia. The objective of this conference was to explore "issues of sexual health, sexuality and sexual behavior amidst emerging gay-identities in South Asia" and provide sexual health prevention services for gay-identified men and MSMs [37]. In the same year (1994), Naz Project with mediation of Khan established Naz Foundation (India) Trust in New Delhi, whose mission along the lines of Naz Project was to implement HIV/AIDS prevention programs among LGBT communities, and act as a technical and financial support providing agency for local NGOs. In 1996, Naz Project evolved into two separate organizations, one continuing the work of Naz Project in London (and was thus named Naz Project London), the other, Naz Foundation International (NFI) with a specific remit to work with MSM population in South Asia. In 2000, NFI registered its liaison office in Lucknow. Over the years, NFI has played a key role in India, Pakistan, Bangladesh and Nepal to develop local MSM community-based organizations to provide HIV prevention, care and support services and develop peer-networks. "Since 1996, NFI has developed or assisted in the development of" some important MSM/LGBT organizations in India such as Bharosa Trust (Lucknow), Gelaya Trust (Bangalore), Manas Bangla (Kolkata), Mirthrudu (Hyderabad), Mitr (New Delhi), Marup Ploi (Imphal), Pratyay Gender Trust (Kolkata), Sahodaran (Chennai), Udaan Trust (Mumbai/Pune) and others. In addition, NFI supports NIPASHA, a national network of MSM HIV-positive groups in Andhra Pradesh (*Snehasudha*), Goa (*Naya Zindagi*), Karnataka (*Spandana*), New Delhi (Love Life Society) and Tamil Nadu (*Alaigal*) [45].

**Figure 2:**
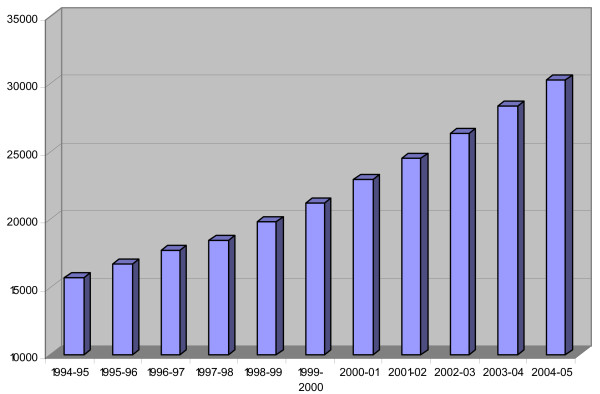
India: No. of FCRA Registered Associations 1994-2005, Source: FCRA Annual Report, MHA, Govt. of India, 2004, 2005

The example of Naz Foundation is only for illustrative purpose here. The basic fact remains that once multinational NGOs entered India and set up their head offices, their primary purpose was to collaborate with indigenous organizations and act as a financial and technical support-providing agency. Thus, the potential availability of a huge amount of international fund catalyzed the mushrooming of NGO-business in every part of the country. Since early 1990s till the end of 2005, international funding for HIV/AIDS in India at current prices has gone up from 19 million to 608 million US dollars. Of this, 313.9 million is National AIDS Control Program (NACP) Phase II funding between 1999–05; $62 million flowing outside National AIDS Control Organization (NACO) between 2004–05; $213.5 million NACP Phase I funding between 1992–99; and $19 million for medium term plan between 1989–92 [46]. It is also estimated that by end 2008, India will have spent about more than a billion dollars for implementing its HIV prevention and care programs alone which includes $608 million available up to the end of 2005; about $400 million available from various international donors in NACP Phase III; and Bill and Melinda Gates' $258 million Avahan Project and USAID Avert Project in Maharastra [46,47]. India's HIV/AIDS transmission is primarily "heterosexual" with more than 84 percent of total transmission taking place through this route, and largely remains concentrated among sex workers, their clients and injecting drug users [46,48-51]. Yet prevention services among MSMs constitute a significant part of NGO programs especially of those working with sexual minorities. Between 1994–2004, the largest number of Gay-Lesbian-AIDS-NGOs was ever registered in the history of Indian subcontinent. Though there is no proper estimate available, data for NGOs that got registered under Foreign Contribution Regulation Act (FCRA) between 1994–2005 reveals that every successive year about 1,459 NGOs were added on an average with 2005 as the single year experiencing largest number (1,970) of NGOs registered [44] (Fig. 2). Not all these NGOs were of "Gay-Lesbian-AIDS-type." From the available data, there is no way to estimate how many NGOs registered under FCRA were of the above category and worse, it is more difficult to know how many of them were in response to HIV/AIDS, considering that individual and collective level efforts also multiplied during the same period. A comprehensive list of LGBT-NGOs established during this period and working on AIDS prevention seems to be unnecessary here. *Humjinsi *(2002) provides a list of such organizations while a more updated list may be available through internet search and in the website of INFOSEM (India Network for Sexual Minorities) and Indian Men's Sexual Health Survey [52,53].

Three "hot topics" among donors were HIV/AIDS prevention, promoting sexual health and sexual rights, and reproductive health. This is because AIDS discourses largely produced India as a "sexually repressed" and "sexually tabooed" society wherein HIV spreads faster than western societies [50,54-56]. Hence, Indians must be made comfortable to their own sexuality to discuss sex openly, without discomfort, so that new HIV infection reduces [57]. Thus to be eligible for getting a fund, say from McArthur Foundation or Bill Gates Foundation, one must promote sexual rights and work with marginalized communities such as queers, sex workers, or drug users. The priority of donors first catalyzed new NGOs being registered with exclusive focus on donor agenda, for example, Sangama (a Sanskrit word for *intercourse*), Social Welfare Association for Men (SWAM), Swabhava Trust, Lakshya Trust, Sangram-Vamp (a sex workers' collective), Aasra Charitable Trust, Gelaya, Sahodaran and others. In many cases, donors helped establish new NGOs that broadly carry forward and implement their mission/agenda. Though examples are numerous, I only cite here two examples of TARSHI (Talking About Reproductive and Sexual Health Issues) and Sangama that were conceptualized and developed by two-year individual fellowships from McArthur Foundation. Grassroots AIDS activism was professionalized by instituting "best practice awards" for NGOs; awards for journalism, HIV-reporting, media fellowship for study abroad; fellowship for HIV medicine, care; and funding for producing documentaries and films, etc. This professionalization opened up new possibilities for unemployed educated youth coming out of universities, researchers and doctors to be absorbed in an ever-expanding AIDS-sector. Retired government officials, high profile bureaucrats got absorbed as Regional Directors of UNAIDS or as senior level managers with international NGOs. Unemployed rural youth were absorbed as peer educators, outreach workers or other low-level jobs. HIV-positive individuals got employment and their families got support.

So lucrative was the AIDS sector, that there has been a cross-sectional mobility from government jobs to the NGO sector. In India, government job still remains one of the important criteria for marriage! I have known many persons individually, who left secured jobs with the Government of India from prominent medical colleges to join the WHO; or from prominent universities and research institutions to join the World Bank or UN systems, simply because the government jobs were too "frustrating with low salary and little possibility to fly business class and stay in a five star hotel abroad." Though personal experience as this may not give a complete picture of the NGO sector, yet, in the absence of proper data on cross sectional mobility of workers, personal experience of close interaction with colleagues as above, might help conceptualize overall professionalization of grassroots activism. Second, the donor-agenda changed the NGO agenda and most of the earlier established NGOs started working on sexuality and AIDS prevention, albeit their mission was to promote education or working on environment, and forestry. Such a shift is more noticeable for grassroots level NGOs that depend on donor support for their survival. However, large NGOs as Population Foundation of India (PFI), the Principal Recipient of Global Fund Round 4 ($18.2 million) and Round 6 ($7.9 million) grant money also reoriented their focus with changing donor priorities. For over 30 years, PFI had been working on family planning and (later) reproductive and child health *without *an HIV/AIDS component in it until 2004 (that was nearly after two decades of the epidemic in India). PFI entered into HIV-business only in late 2004 with its initiation as the principal recipient of the Global Fund Round 4 grant.

Once the agenda is clear, then follow the methods of implementation. In almost all cases, program inputs were juxtaposed from different contexts. Toolkits, handbooks, guidelines, strategic plan, resource materials, training manuals, virtually every truths and norms about programs were imported from donor's home country. In the name of providing technical support and capacity building, a gospel of Western truths and norms about development was pumped into NGO programs, a trend that has been well documented from various contexts at various times especially in India and Latin America [14,58-60]. Thus queer film festivals, gay pride parades, queer chatrooms, queer advertising, queer films, queer networks, support groups, queer NGOs, and queer reporting were all instituted as program strategies. My experience from project design workshops of a few NGOs reveal that many considers LGBT *Pride Parades *as a good program strategy for reducing stigma. Thus, *Calcutta (Kolkata) Gay Pride Parade *has become a yearly event since 2003 in which activists from all over India as well as from other countries participate in street march followed by a weeklong program of film screening, workshops, book reading, seminars, etc. [61]. Legalizing prostitution is advocated by many powerful groups as a magic solution to HIV/AIDS (prominent among this group are DMSC, Kolkata; Sangram-Vamp, Sangli; and National Network of Sex Workers, New Delhi). NGO-advocacy at the policy level finally culminated in Planning Commission of India's recommendation to the government for legalizing sex work and homosexuality [62,63].

An important component of Indian HIV/AIDS program was "media advocacy," a donor-driven concept to diffuse and popularize the ideas of ruling class to such an extent, that common people perceive and evaluate the social reality in *their *context. Hence, popular media was targeted to feature stories, articles, news, proceedings of workshops/conferences etc. by preferential treatment (such as free media-registration for workshops, seminars) and institutionalizing "media fellowships." Through media fellowship, journalists were selected to study in a foreign university and trained in health reporting so that when they return, they could serve a specific function for the donors. Every forum, workshop, seminar these NGOs organize, becomes a platform for magnifying the problem and increasing the number of HIV positive individuals in India [64]. Issues related to HIV/AIDS became a common feature in the mainstream media since mid-1990s, and more so after 1998. Networks of NGOs working on the same issue were also established within and across cities to build up sites of resistances and horizontal integration of power.

Another vital component of NGO programs was "situation assessment" or "community needs assessment" (CNA) usually carried out before starting the program. For conducting CNA, NGO workers went on searching for HIV positive individuals and other vulnerable groups such as gays, eunuchs, and *kothis *perceiving risk on *their *behalf and motivating them to go for an HIV test. This has been reported particularly with HIV positive people and injecting drug users in Delhi [65]. Such an exercise had put two interest groups at stake: first, if a threshold population of HIV-positive individuals, gays and eunuchs were not found, outreach workers lose their job! And second, if "need" is not reflected from such an exercise, NGOs lose their potential funding. Hence "construction" of an agenda and inflation in reporting was inevitable in which more number of target population meant more money for program implementation. The fact that India's HIV/AIDS burden is grossly overestimated is now being revealed only after two decades of unscientific reporting and methods of estimation coupled with a general hype, hysteria and biases among NGO workers and government officials. For example, early in the 1990s (1986–91), it was reported that 300 Indians contract HIV every day [57] (p. 14–15), that rose to 1,400 by 1999 and 5,000 by 2002 [66] (p. 115). Similarly, many individuals and institutions projected India's total number of AIDS cases with grossly misleading figures. In 2001 for example, while the government claims that there were about 4 million total AIDS cases, some statisticians report it as 19 million that is expected to rise to 62 million by 2016 [67]. Similarly, UNAIDS and CIA estimates suggest that India will have 20–25 million infection by 2010 [68]; World Bank estimates it as 20 million by 2005 [66] (p. 113); yet some other estimate suggests it as 50 million by 2025 [69]. The fact that all these were a part of the overall panic with which HIV was treated is now coming into picture with more accurate estimates and population based surveys. For example, a population based survey in Guntur district of South India reveals that "sentinel surveillance method" to arrive at HIV/AIDS figures overestimates the burden by 2–3 times than population based data [70]. The reasons for this overestimation are due to addition of unnecessary HIV estimates from STI clinics; common practice of referral of HIV positive and suspect patients by private practitioners to public hospitals; and a preferential use of public hospitals by lower socioeconomic strata used in sentinel surveillance method. A more recent and authentic population based data, National Family and Health Survey conducted under international (US) financing and supervision endorses the above fact, reducing India's total HIV infected people from 5.7 million to 2–3 million (0.9 percent to 0.3 percent of adult population) [71]. Similar trend is observed in Africa where population based surveys have led the United Nations to gradually reduce the estimated number of infected people country by country. For example, when Kenya was carefully surveyed in 2004, its prevalence rate dropped by more than half from 15 percent UNAIDS' estimate of 2001 to 6.7 percent (*ibid*.). As Daniel Halperin, an HIV-expert at the Harvard School of Public Health says: "If the total number of cases in the world is half of what you've been saying, that's a bitter pill to swallow... AIDS-fighting agencies have such a stake in portraying the epidemic as an approaching Armageddon that they are hesitant to make significant downward revisions in estimates... So every year they lower the numbers a little bit, and retroactively change the estimates of what it used to be" [c.f. [71]]. If Halperin is correct, then the general suspicion about India's inflated number of HIV-positive individuals seems to be valid.

The death of a gay-rights activist Siddhartha Gautam in 1994, a young lawyer who was instrumental in preparing the report *Less Than Gay*, led to the establishment of a yearly film festival in his memory and organized by an informal group called Friends of Siddhartha [72]. Films on LGBT issues and HIV/AIDS were shown in European cultural centers in Delhi attended by NGO workers, gay network members and support groups. Another film festival that was formally instituted in 2003 was *Larzish: International Film Festival of Sexuality and Gender Plurality *and funded by international donors such as Astraea Lesbian Foundation for Justice, HIVOS, Mama Cash and their local partners in India such as LABIA and Humsafar Trust [73]. Since Bollywood did not produce any mainstream lesbian and gay film until 2004, most of the films that were shown in these film festivals were foreign films primarily attended by English educated elites. It is unclear what relevance these foreign films had to the issues affecting marginalized queer communities vulnerable to HIV/AIDS in India, other than implying that anything could be picked up from different locations and fit into any social or cultural context as diverse as India and US.

So far, I have only examined one among many other causes of LGBT mobilization in India that is intimately linked to HIV/AIDS funding. This is not to argue that individual and collective level efforts do not exist. There are many such efforts ongoing in different parts of the country and many of them operate without any funding whatsoever, and without the context of HIV/AIDS epidemic. Some of these initiatives could be Nigah Media Collective, Prism, Rod Rose, Anjuman or Voices Against 377. Moreover, it must be acknowledged that efforts made by LGBT organizations have resulted in relatively low HIV prevalence among MSMs in India. As National AIDS Control Organization's *2005 HIV/AIDS Epidemiological Surveillance and Estimation Report *indicates, the prevalence of HIV infection among MSMs has gone down from 12 percent in 2003 to 8.7 percent in 2005 (p. 4).

#### 2.3. The "homophobic" State and its reaction

Now I turn to examine the second part of my argument that queer mobilization as mediated by globalization and AIDS epidemic has simultaneously strengthened "homophobic" discourses of heterosexist nationalism in India. "Homophobia" as conceptualized by George Weinberg in 1971 and popularized through his book *Society and the Healthy Homosexuals *has received much criticism from opponents. Antigay critics, for example, former US Congressman William Dannemeyer complained that homophobia shifts the terms of debate away from the idea "that homosexuals are disturbed people by saying that it is those who disapprove of them who are mentally unbalanced, that *they *are in the grips of a *phobia*" [74]. Gregory Herek thus considers *homophobia *as a word bearing negative connotation and there is need to advance a new vocabulary and scholarship in this area. Herek notes that homophobia has served as a model for conceptualizing a variety of negative attitudes based on sexuality and gender, and derivative terms such as *lesbophobia, biphobia, transphobia *etc. have emerged as labels for hostility toward sexual minorities. Though society has negative attitudes toward homosexuals, minimal data available do not support the claim that most antigay attitudes represent a true *phobia*. Thus, a more nuanced vocabulary is needed to understand the psychological, social, and cultural processes that underlie the oppression. Herek prefers using words such as *sexual stigma*, *heterosexism*, and *sexual prejudice *instead of *homophobia*. Since homophobia remains a contested notion, I use it in this paper within an inverted comma.

The first AIDS case in India was detected in Chennai in 1986. Considering it immediately as a "foreign disease," the government adopted a repressive AIDS Control Policy (1989) through which it outlined "contact tracing," testing of sex workers, injecting drug users, and other high-risk groups and adoption of a *quarantine *approach if found HIV-positive to protect larger population at risk. Consequently, sex workers, drug users and MSMs were forcibly tested and jailed for several months in Chennai, Mumbai and Goa. For example, in 1989, Dominic de Souza, a World Wildlife Fund employee and a gay on whose life Bolllywood film *My Brother Nikhil *(2005) was produced, was kept in a solitary confinement for over a month by Goa government. Similarly, Tamil Nadu government forcibly tested several hundred sex workers in 1990 and then locked up 800 infected women for several months [57] (p. 27). Such a social control approach seems to have worked in small populations with strict centralized ruling and strong Soviet-style policing as in Cuba; but India had nothing in common with Cuba's universal literacy, excellent health care delivery and a frank sex education campaign in schools to adopt this policy (*ibid*., p. 30). Due to sustained activism of indigenous human rights groups and pressure from the World Bank, India withdrew its National AIDS Control Policy of 1989 as a "condition of loan" for implementing its National AIDS Control Program and adopted a liberal, rights based perspective for prevention and control of AIDS [[57], p. 86; [66], p. 118]. For developing this new rights-based policy, technical support was imported from abroad and organizations like WHO helped India developing such a policy. Thus though sex work, drug use and homosexuality remained criminalized, "targeted interventions" were launched among "high-risk groups" across many cities. The government adopted a double-standard of morally and legally disapproving despised sexualities, but simultaneously funding collectives of sex workers and MSMs for implementing national HIV/AIDS prevention programs.

On July 7, 2001, police in the city of Lucknow, Uttar Pradesh, raided a park that was frequented by MSMs. The raid was based on a complaint filed by a person who alleged that he had been sexually assaulted while providing massage service in the park. Taking this queue, police raided the offices of Bharosa Trust and Naz Foundation International, two NGOs working with MSMs under the charges of running a "gay-club" and a "call-boy racket" in the city with the pretext of imparting HIV/AIDS awareness programs. The Project Manager of Bharosa and the Director of Naz along with four outreach workers were arrested on charges of propagating and indulging in "unnatural sex" under Section 377; Section 292 (sale of obscene books); Section 120b (criminal conspiracy); Section 109 (abetment) of the IPC; Section 60 of the Copyright Act; and Section 3 and 4 of the Indecent Representation of Women Act. The basis for such a charge by police was that during the raid in NGO-premises, they found condoms and lubricants (for aiding in "unnatural sex"); communication materials (termed as "pornography"); dildo used for condom demonstration (termed as "sex toy"); and video cassettes and photographs (termed as "obscene literature") [75]. The offices of Naz Foundation and Bharosa Trust were sealed. During the raid, police ignored all other reports and documents shown to them to establish that the organizations were working under the purview of NACO-policy. Instead, they went on justifying the arrest and spread misinformation in popular media claiming that they wanted to stop the "vice of homosexuality." The NACO and Uttar Pradesh State AIDS Control Society chose a policy of silence: where a public statement saying that these two organizations were working under the purview of their policy could have saved sufferings of the four arrested, they silently watched the four ending up in jail for 47 days (*ibid*.).

A few days after the Lucknow incident, NGOs working in the field of HIV/AIDS came together in New Delhi to form an alliance of organizations whose primary purpose was to defeat and repeal the very section of IPC 377 under which two NGOs were arrested. Two prominent members of this alliance were Naz Foundation India Trust and Lawyer's Collective. The alliance took over the case of challenging the constitutional validity of Section 377 of IPC through public petition (once filed by ABVA in 1994). Towards late 2001, Naz Foundation on behalf of the petitioner filed a public interest litigation (PIL) in Delhi High Court [76]. The foundation argued that the penal code provision not only violates right to life and liberty as outlined in the Indian Constitution but also impedes effective control of AIDS. In its petition, the group asserts that Section 377 is discriminatory because it criminalizes predominantly homosexual acts and imposes traditional gender stereotypes of *natural *sexual roles for men and women upon sexual minorities. In effect, Section 377 provides moral and legal sanction for the continued social discrimination of sexual minorities (*ibid*.).

Towards early January 2003, Delhi High Court ordered the Indian government to respond within a month and clarify its stand on the PIL filed by NAZ Foundation seeking an end to the law that makes homosexual relations a crime [77]. The government (Ministry of Home Affairs) in its affidavit submitted to the Delhi High Court responded that, "the basic thrust in the argument of pro-gay activists is the perceived violation of fundamental liberty guaranteed in Article 19 of the Constitution of India. However, there is no violation of fundamental liberty as long as any act of homosexuality/lesbianism is practiced between two consenting adults in privacy as in the case of heterosexuality" [78]. The Affidavit said that in India, Section 377 has been basically used to punish sexual abuse to children and to compliment lacunae in rape laws. It has rarely been used to punish homosexual behavior. For example, in the entire history of statute from 1860 to 2002, there was only 30 reported cases under Section 377 that came before various High Courts and the Supreme Court since 1830. The large majority of prosecutions were due to non-consensual acts of sodomy, with only 4 cases where consensual acts of sodomy have been brought to court, 3 of which are prior to 1940 (pre-independence India). In addition, 50 percent of total cases consist of sexual assaults committed on minors, whereas only 5 out of 30 being on adults [17,79]. Such facts indeed pose a question on the practicality and need to have such a law that has rarely been used. The affidavit also mentioned that the provision becomes operable "only when there was a report to the police for either sodomizing or buggering." Such an explanation barely justifies the government's stand for retaining Section 377, as lacunae in rape laws could always be filled-in by including child sexual abuse or non-consensual sodomizing as suggested by the Law Commission of India in its 172^nd ^Report.

Home Ministry affidavit also said that there was no tolerance of such a practice in Indian society. Legal conception of homosexuality is not independent of society. "Public tolerance of different activities changes over time and the legal categories get influenced by those changes... Acts, which have been glorified in the past, like dowry, child marriage, domestic violence, widow re-marriage etc. have now been brought under the preview of criminal justice. Therefore, changes in public tolerance of activities *lead to campaigns *to either criminalize some behavior or decriminalize others... While the Government cannot police morality, in a civil society, criminal law has to express and reflect *public morality *and concerns about *harm to the society *at large..."(*ibid*., emphasis mine).

The government thus believes that public morality is upheld and maintained by penalizing "unnatural" sexual acts *"with any man or woman" *through Section 377. The interesting point is, how could the government certify public morality on "unnatural sex" when various national level surveys indicate the opposite? For example, successive surveys conducted by India Today-AC Neilson and ORG-MARG in 2003 (covering 2,305 unmarried, married and separated women between 19–50 years across 10 cities); 2004 (covering 2,499 married and unmarried men between 18–55 years across 11 cities); 2005 (covering 2,035 single women between 18–30 across 11 cities); and 2006 (covering 2,559 men of 16–25 years across 11 cities) reveal that 37 percent single young men have had a homosexual experience in 2006 compared to 31 percent in 2004 (*India Today*, November 13, p. 37); whereas 3–5 percent women reported having lesbian experience in 2005 (*India Today*, September 26, p. 47). Similarly in 2005, 28 percent single women have tried anal sex while another 8 percent have tried bisexual sex (*India Today*, September 26, p. 46). Women reported experiencing anal sex in 2005 is significantly higher than 2003 level, which was 13 percent (*India Today*, September 15, p. 46); whereas men reported having tried heterosexual anal sex is as high as 32 percent and bisexual sex as 11 percent in 2006 (*India Today*, November 13, p. 60). Though this is not a nationwide survey with representative sampling and there could be sample bias, these figures only go on to tell that the "public morality" government is concerned about has little practical ground as people are already having "unnatural sex" criminalized under Section 377. The government also fails to recognize that the current PIL is indeed a part of broader "campaign" for decriminalizing consensual adult sexual act. Instead, it goes on arguing, "even assuming that acts done in private with consent do not in themselves constitute a serious evil, there is a *risk *involved in repealing legislation which has been in force for a long time..." (*ibid*.). Again, no reference to the perceived "risk" is provided in the affidavit, other than adamantly arguing that a colonial legacy needs to be maintained since it's been here with us for a long time!

Yet based on these misleading statements by government, the Delhi High Court in its ruling on September 2, 2004 dismissed the petition on ground that the petitioner has no *locus standi*, meaning there was no "cause of action" in the petition since no prosecution is pending against the petitioner. "Just for the sake of testing the legislation, a petition cannot be filed... the court does not express opinion when nobody is really aggrieved by the action which is impugned and does not examine merely academically the impugned action of the legislature or the executive. In view of the above, we feel that an academic challenge to the constitutionality of a legislative provision cannot be entertained. Hence, the petition dismissed" [80].

Naz Foundation then filed a Review Petition against the Court order, which was also dismissed in a ruling on November 3, 2004. A Special Leave Petition was then filed with the Supreme Court of India on the limited question of whether the Court could dismiss the petition on ground that it was purely "academic" and there was no "cause of action." The Supreme Court in its ruling on February 3, 2006 referred the case back to Delhi High Court contending that the Court had erred in rejecting the original petition that Naz Foundation had no *locus standi *[81]. One of the respondents, the Union of India, submitted that the petition against Section 377 was of public importance and merited examination. The Supreme Court also allowed the petitioner to seek an expeditious hearing as the matter has been pending for a considerably long time. Even NACO on behalf of the respondents agrees in its Affidavit dated July 17, 2007 that "enforcement of section 377 can adversely contribute to pushing the infection underground, make risky sexual practices go unnoticed and unaddressed. The fear of harassment by law enforcement agencies leads to sex being hurried, leaving partners without the option to consider or negotiate safer sex practices" [82]. NACO Chief, Sujatha Rao has agreed in public speeches that this law as "hateful, not acceptable, anachronistic, and scrapping the law is fundamental" to the fight against AIDS [83].

#### 2.4. "Homophobia" and the language of resistance

The Humsafar Trust, immediately after its establishment in 1994 proposed to hold the First South Asian Gay Conference in Mumbai. Objecting this move, the Vice President of National Federation of Indian Women, a women's organization affiliated to the Communist Party of India, through a widely endorsed letter appealed to the Prime Minister to cancel permission to host the Gay Conference [84]. Describing it as an "invasion of India by decadent western cultures and a direct fall-out of our signing the GATT agreement," it urged the Prime Minister "not to follow Bill Clinton's immoral approach to sexual perversions in the US" and to immediately cancel the permission to hold the Conference [85]. However, the Conference indeed took place with about 70 participants and received positive media attention.

In 1998, Deepa Mehta's film *Fire *got nationwide release. The story of *Fire *revolves around lesbian relationship of two unhappily married women of the same family (named after Hindu goddesses Sita and Radha worshipped all over the subcontinent). On its opening day in India, Right wing Hindu nationalist group destroyed movie theatres in protest against its lesbian storyline. Theatre halls in many cities such as Mumbai, Surat, Lucknow, New Delhi and Kanpur were stormed, destroyed or burnt [86]. The movie was immediately banned in India and referred to the Censor Board for a review while it was off for showing in Pakistan. The banning of the film raised a series of controversy in popular media both in India and abroad [87]. Madhu Kishwar, one of the noted Indian feminists published a comprehensive review of the film *Fire *in women's magazine *Manushi*, arguing how the West views and interprets culture and tradition of the East primarily through a Eurocentric lens. Branding the film as a crude caricature of Indian culture and tradition, Kishwar, in her review argued that:

"...by crudely pushing the Radha-Sita relationship into the lesbian mould, Ms Mehta has done a big disservice to the cause of women... In most Indian families, even when sexual overtones develop in the relationship of two women situated as are Radha and Sita, no one generally gets upset about it provided people don't go around flaunting their sexual engagement with each other... Given that in a gender segregated society like ours, women spend a lot more time with each other than they do with men, such close bonding is fairly routine. Indians, by and large, are not horrified at witnessing physical affection between two people of the same gender. Two women friends or female relatives sleeping together in the same bed, hugging, massaging each other's hair or bodies is seen as a normal occurrence and even encouraged in preference to similar signs of physical affection between men and women. Such physical affection between women is not ordinarily interpreted as a sure sign or proof of lesbian love... [However after being] exposed to this controversy, women will learn to view all such signs of affection through the prism of homosexuality. As a consequence many will feel inhibited in expressing physical fondness for other women for fear of being permanently branded as lesbians" [88].

Kishwar's broader argument in her article was that India offers a favorable social climate for LGBTs by approving of many "homosocial" relations until people "come out" and "flaunt" their sexuality in public, which she thinks is derivative of a country's history, culture and tradition. I think Kishwar had a broader point – the political rhetoric of confession and "coming out" may not have the same effect and acceptance in transitional societies as India or other South Asian countries. Kishwar's argument is strikingly similar to what has been argued elsewhere in other Asian societies. For example, a press release in 1998 Chinese *Tongzhi *Conference in Hong Kong declared that "the lesbi-gay movement in many Western societies is largely built upon the notion of individualism, confrontational politics, and the discourse of individual rights. Certain characteristics of confrontational politics, such as coming out and mass protests and parades, may not be the best way of achieving *tongzhi *liberation in the family centered, community-oriented Chinese societies... In formulating the *tongzhi *movement strategy, we should take specific socio-economic and cultural environment of each society into consideration" [89].

In 2004, when the first Bollywood lesbian film *Girlfriend *was released, Hindu Right activists forcibly stopped screening of the film, hurled stones breaking the glass-panes of the cinema halls, shouting slogans and staging protest demonstrations across various Indian cities including Mumbai, Varanasi, Indore, Bhopal and Nagpur [90]. The ruling Bhartiya Janta Party (BJP) demanded a review of the film by the Censor Board and deletion of scenes which were "objectionable and against Indian culture." The BJP spokesman, Mukhtar Abbas Naqvi, said, "the film should be reviewed and shots which are objectionable and against Indian culture should be removed. The film does not mirror the realities of Indian society (*ibid*.)." Homosexuality is thus seen by Hindu nationalists as "*un-Indian, alien, imported *from the West and a *vice *of British colonialism." Based on several internal publications of BJP, RSS and Shiv Sena, Paola Baccheta (1999) has argued that one of the pillars of Hindu nationalism rests on "queerphobia" in which queer gender and sexualities are constructed outside the Hindu nation (and hence must be exiled!) through a misogynist conception of gender and heterosexist notion of sexual normatively [24]. Naqvi's statements clearly corroborate Baccheta's claim.

Even within Left camp, sexual politics is received with strong disapproval. For example, in 1996 when *Economic and Political Weekly *(February 3) carried an article on *Gay Rights in India *by Vimal Balasubrahmanyan, there was strong opposition from a Marxist thinker, H. Srikanth. Terming sexual identity politics as "backward and reactionary" just like Sati, polygamy and caste system, Srikanth goes onto argue that gay liberation movement is imported from the western decadent bourgeoisie. He states that:

"...the justification of homosexuality as a normal behavior is based on the assumption that anything based on mutual consent and not aimed at harming others is acceptable and permissible. This assumption is based on liberal bourgeois notion that a person is free to do anything as long as he does not touch another's nose. To interpret what is normal for individual is also normal for the society is to fall into the trap of bourgeois individualism which reduces society to a sum total of separated and unconnected individuals. If coming out of compulsory heterosexuality is possible, I don't see any reason why an individual can not come out of homosexual relations that too in a system where monogamous relations cease to be discriminatory and oppressive" [91].

Srikanth thus argues that heterosexuality is not only *natural *but also *compulsory *and coming out of homosexuality is both possible and desirable. He also fails to conceptualize that once a person comes out of homosexuality, what remains is a compulsory heterosexuality thus ignoring the "power" that operates through heteronormativity, and resistance offered by "coming out." Contrary to Srikanth's self-proclaimed "official" Marxist position on homosexuality, Brinda Karat, General Secretary of the Communist Party affiliated All India Democratic Women's Association, wrote in a strongly worded letter to the Law Minister Arun Jaitley, that "the government does not have a *locus standi *to interfere in private sexual activity of two consenting adults and hence Section 377 of IPC must be scrapped" [92].

### 3. Implications for programs

After broadly reviewing the social and political circumstance under which LGBT identity politics operates in India, let me now clarify what is the implication of a donor-induced LGBT identity politics within the context of HIV/AIDS. Many LGBT-rights activists (including academics) have contended that marriage and family as institutions come on the way of people's "coming out" process, and the familial pressure for marriage in India do not allow individuals to "come out" as gays/lesbians [4,21,32,33,38,93]. Thus due to familial pressure, they lead a "double life" as bisexual (*ibid*.) with "repressed" sexualities. Such an explanation seems to be oversimplistic, as it does not consider all the social and political implications of "coming out" in a transitional "homophobic" society. While "coming out" may be a politically empowering option, it remains unclear how "homophobia" inherent within family could be dealt with or whether it would be a desirable alternative to take a "queer" out of family to declare himself as "gay." Much less it captures, if people are living as bisexuals within marriage, then whether promoting divorce would be a desirable program strategy for donors to let people develop their sexual identity independent of the familial control! "Confession," in the same political rhetoric of the West, may create more deepening social and political problem of "homophobia," cultural nationalism, and fascist resistance in Eastern societies, including loss of psycho-social and economic support structures for "out" gays. However, one may always argue the other way that "coming out" in a globalizing world may actually enhance economic opportunities if one happens to belong to the privileged lot of urban, educated elite.

Second, a donor-induced LGBT identity politics also leads to globalization of categories. As Shannon Woodcock pointed out that "freeing" the pre-existing categories of sexual identities from repressed social positions, could be read as a "movement of containment." Through defining traditional sexual practices as politicized LGBT identities, "the existing multiplicities of sexual practice and ways of performing them in society are formalized in new western categories with their specific place in an international political trajectory. In order to form these new communal identities, individuals are urged to participate in the self-perpetuating western culture of *confessing*"[9], that creates a new set of organizing sexual identities damaging the existing, more subtle ones. What is being globalized, thus, is an American version of confrontational queerness without recognizing the social and political structure of Eastern societies. In India, barring a few NGOs, existing multiplicities of queer sexualities such as *hijras*, *kothis*, *kinnars*, *panthis*, *jogtas*, *dangas*, *alis*, *double-deckers*, *chhakkas*, and *dhuranis *are commonly clubbed together by HIV/AIDS activists as LGBTs thus redefining existing sexual identities/practices in a predefined Western mould of "performance."

With specific reference to HIV/AIDS, donor emphasis on "sexual" (hetero/homo) routes of transmission ignores other important non-sexual routes. In a recent article, Gisselquist and Correa (2006) argue that "overestimating the contribution of commercial sex to India's HIV epidemic misleads prevention programs to ignore other risks, and promotes the stigmatizing assumption that HIV infection is a sign of immoral behavior" [94]. With best and highest plausible evidence-based estimate, they argue that female sex workers and their clients account for 2–15 percent of total HIV infection among adults, far less than 44–68 percent reported by model-based estimates. According to them, HIV prevention focusing on "high risk groups" (consisting of sex workers, clients, MSMs and drug users) has dominated India's programs for over two decades whereas non-sterile medical injections and other risky blood exposures in health care and cosmetic services account for an important proportion of HIV infection. The moral of the story is: sexual route (homo or hetero) has received undue attention and emphasis from donors than an important route of infection, that is not "sexy" to talk about and that does not bring much money into HIV/AIDS funding-machine. The present LGBT activism must be viewed within this context. Many NGOs and health activists have already expressed concerns that donors are distorting India's health priorities by excessively focusing on HIV/AIDS [95].

## Conclusion

In Deepa Mehta's film *Fire *(1998), Sita, while convincing her lesbian-lover (sister-in-law) to break away from her marital relationship and run away with her tells the following dialogue: "...there is no word in our language that can describe what we are or how we feel for each other..." It is indeed surprising that India, a country with 18 constitutionally recognized languages and nearly 2000 dialects, has no equivalent word for the "lesbian." There could be at least two possible interpretations of the above dialogue: first, that same sex love and sexual relationships, though exist in a traditional society like India, her language, culture and tradition is incapable of expressing the modern form of sexual identity categories; and second, Sita also implies that in the West, we would be called as "lesbians" but the sheer absence of its equivalent word in India does not recognize the existence of lesbian relationships and their rights. Hence a movie like *Fire *needs to be produced to "liberate the *sexually repressed *Indians" by introducing them to the "recently discovered, fashionable, western versions of sexual freedom" and identity [88] (original emphasis added).

Even today, no equivalent word exists for the term "homophobia" in any of the Indian languages, though Indian society remains highly "homophobic." This "homophobia" is blamed on to the British who introduced it in India by enacting a law in 1861, IPC 377. Scholars argue that before the introduction of this law, Indian society was much tolerant to the issue of homosexuality [32]. Other scholars have argued, that the systematic silence of ancient medieval and modern Indian literature on homosexuality reflects the conservative sexual mores of people [40]. In the voluminous body of literature that is produced in South Asian countries, in English as well as in about twenty indigenous languages, there is hardly an imaginative text that sympathetically explores the theme of homosexuality.

India is a highly gender segregated society. Free mixing of sexes is not allowed especially after one attains puberty. In many parts of rural north India, girls are withdrawn from school with the fear of mixing with opposite sex [96]. In such a society, a person spends much time with members of the same sex and having friendship or emotional attachment in such relationship is quite common. Even when sexual relationship develops within such friendships, nobody goes on displaying their sexual engagement publicly or prefer "coming out" of the family to assert their individual liberty and rights. In this social context, same sex friendship and spaces are generally more approved of by parents than opposite sex friendship and mixed gender space [38,88]. Thus many *homosocial *behavior such as sharing a bed, body messaging, hugging or kissing between same sex members is not interpreted as homosexual relationships.

The important point is, sexual diversity, gender plurality, sexual rights and freedom must be preserved and upheld in diverse societies in their own way. This ought to be the spirit of a "rights based approach" – leaving indigenous queer sexualities perform their own way as they have done so since generations. One should not interpret this as equal to maintaining a hegemonic social structure in which sexual minorities are repressed. Social justice is social justice and the pursuit of it must be the goal of a democratic nation-state. On the other hand, it is erroneous to argue that societies where sexual minorities are not politically organized as LGBTs, necessarily repress queer cultures. India has had a beautiful system and diverse ways of performing and integrating queer sexualities within its social structure. Donor-induced mobilization, and baptization of traditional sexual minorities into a globalized LGBT identity category blurs sexual diversities, sexual cultures, and contain the strategic dynamism with which indigenous queer sexualities perform, relate and live in societies. LGBT identities may emerge in Eastern societies in different ways and *without *the political rhetoric of the West that recognizes the interrelationships of social, political, economic and cultural structures far from a linear progressive model toward Western-style queerness. As Eduardo Nierras puts it, "when we say to straight people, or more rarely to Western people, *we are like you*, we must remember to add, *only different*"[7].

## Competing interests

The author(s) declare that they have no competing interests.
